# Virtual global health in graduate medical education: a systematic review

**DOI:** 10.5116/ijme.62eb.94fa

**Published:** 2022-08-31

**Authors:** Lisa Umphrey, Nora Lenhard, Suet Kam Lam, Nathaniel E. Hayward, Shaina Hecht, Priya Agrawal, Amy Chambliss, Jessica Evert, Heather Haq, Stephanie M. Lauden, George Paasi, Mary Schleicher, Megan Song McHenry

**Affiliations:** 1 Department of Pediatrics, University of Colorado School of Medicine, Aurora, Colorado, USA; 2 Case Western Reserve University School of Medicine, Cleveland, OH, USA; 3 Cleveland Clinic Lerner College of Medicine, Case Western Reserve University School of Medicine, Cleveland, OH, USA; 4 Department of Pediatrics, University of Utah, Salt Lake City, UT, USA; 5 Department of Pediatrics, Indiana University School of Medicine, Indianapolis, IN, USA; 6 Mid-Atlantic Permanente Medical Group, Washington, DC, USA; 7 Child Family Health International, El Cerrito, California, USA; 8 Department of Pediatrics, Baylor College of Medicine, Texas, USA; 9 Nationwide Children's Hospital, The Ohio State University, Columbus, OH, USA; 10 Mbale Clinical Research Institute, Mbale, Uganda; 11 Cleveland Clinic Floyd D. Loop Alumni Library, Cleveland, OH, USA

**Keywords:** Global health, education, graduate medical education, virtual, pandemic

## Abstract

**Objectives:**

To synthesize recent virtual global health education activities for graduate medical trainees, document gaps in the literature, suggest future study, and inform best practice recommendations for global health educators.

**Methods:**

We systematically reviewed articles published on virtual global health education activities from 2012-2021 by searching MEDLINE, EMBASE, Cochrane Library, ERIC, Scopus, Web of Science, and ProQuest Dissertations & Theses A&I. We performed bibliography review and search of conference and organization websites. We included articles about primarily virtual activities targeting for health professional trainees. We collected and qualitatively analyzed descriptive data about activity type, evaluation, audience, and drivers or barriers. Heterogeneity of included articles did not lend to formal quality evaluation.

**Results:**

Forty articles describing 69 virtual activities met inclusion criteria. 55% of countries hosting activities were high-income countries. Most activities targeted students (57%), with the majority (53%) targeting trainees in both low- to middle- and high-income settings. Common activity drivers were course content, organization, peer interactions, and online flexibility. Common challenges included student engagement, technology, the internet, time zones, and scheduling. Articles reported unanticipated benefits of activities, including wide reach; real-world impact; improved partnerships; and identification of global health practice gaps.

**Conclusions:**

This is the first review to synthesize virtual global health education activities for graduate medical trainees. Our review identified important drivers and challenges to these activities, the need for future study on activity preferences, and considerations for learners and educators in low- to middle-income countries. These findings may guide global health educators in their planning and implementation of virtual activities.

## Introduction

Global health (GH), a rapidly growing field focused on advancing international and interdisciplinary healthcare^1^^-^^12^ while addressing health inequities,^13^ is an increasingly common component of graduate medical education and international partnerships.^1^^,^^14^ The COVID-19 pandemic disrupted in-person GH education (GHE) activities such as international clerkships and rotations^15^^, ^^16^ and worsened inherent inequities in GH.^17^^, ^^18^ Typical challenges encountered in GHE work, including distance, communication, and barriers to bidirectional exchange of staff and learners 6 worsened throughout the pandemic,^19^^, ^^20^ highlighting the need for thoughtful development of virtual GH curricula and practice.

Since the start of the pandemic, much has been published on shifting graduate medical education activities into the virtual realm, but little research focuses on virtual approaches to GHE, particularly within GH partnerships where barriers such as poor internet access persist.^14^^, ^^21^^-^^23^ While several papers discuss the use of virtual education for GH preparation, simulation, and education,^7^^, ^^24^^-^^29^ ethical considerations in GH engagement,^30^^-^^32^ and clear learner competencies for GHE within GH partnerships,^1^^, ^^24^^, ^^25^^, ^^27^^, ^^28^ limited guidance exists regarding methods to virtually sustain or improve formerly in-person GHE activities during the pandemic or similar disruptive global challenges. Few previous papers focus on supporting partners in low- to middle-income countries (LMIC) during times of crisis,^26^^, ^^33^ and it is unclear how GH competencies can be reinforced virtually for learners in high income countries (HIC) while prioritizing the needs of partners in LMIC.^20^^,^^21^^,^^34^^,^^35^ Last, to our knowledge no current studies examine faculty or learner preferences for virtual GHE activities (VGHEAs).

Virtual GH content is necessary and relevant now due to current travel restrictions, but this mode of engagement will undoubtedly be a key component of GHE moving forward. 15 Hindrances from financial constraints, ongoing travel restrictions, threats of future COVID-19 variants, and equitable access to vaccination may continue to limit in-person GHE activities.^19^^,^^20^ VGHEAs may provide the GH community with lower cost, more attainable engagement strategies, and may facilitate mutual learning, goal setting, and problem solving.

There is a crucial need for evidence about VGHEA planning, implementation, and continuation, particularly regarding the specific needs of learners in LMICs, to guide GH educators and the creation of GH programming. This systematic review, therefore, aimed to identify and synthesize recent VGHEAs (including their enablers and barriers) targeting health professional trainees of any level, to document gaps in the existing literature, to identify areas of future study, and to contribute to preliminary foundational data to inform future best practice recommendations for GH educators.

## Methods

We used the Preferred Reporting Items for Systematic review and Meta-Analysis (PRISMA) Protocols 2015 Checklist^36^ to perform our systematic review, which we chose as the most appropriate methodology to summarize recent VGHEAs over our review period. We registered the general systematic review protocol with PROSPERO on February 14th, 2021.^37^ Ethical approval was not required for our review.

### Eligibility criteria

Inclusion in this review required that articles from the primary literature between 2012-2021 focus on existing and sustained GH curricula, programs, activities, or online content. Our definition of “GH content” included any activity highlighting health disparities due to resource level, geography, or access to care. The administration of the GH content had to be primarily virtual, not supplementary to an in-person activity. The target users of the content had to be health professional trainees of any level or specialty. We chose to include articles between 2012-2021 to focus our evaluation on more recent technology and on articles with more robust descriptions of virtual activities.

Our review excluded online content not otherwise described in the primary literature; general open access resources without a stated objective to reach trainees in under-resourced or LMIC settings; descriptions of telemedicine services; and non-human GH topics. If multiple papers described the VGHEA, our review included only the most recent article. Our review also excluded Project ECHO (Extension for Community Healthcare Outcomes) 38 discussions, as they are not trainee-focused and were outside the scope of this manuscript. Please see Appendix 1 for full inclusion and exclusion criteria.

### Search strategy

A medical librarian (M.S.) constructed a comprehensive search strategy to capture the concept of VGHEAs (Appendix 2). We used the strategy to search the following databases on November 4, 2021: Ovid MEDLINE®, Ovid Embase, Cochrane Library from Wiley, Education Resources Information Center (ERIC, via EBSCO interface), Scopus via Elsevier, Web of Science from Clarivate Analytics, and ProQuest Dissertations & Theses A&I. One co-author (N.E.H.) searched the grey literature sites per the strategy in Appendix 2. Two authors (N.L. and L.U.) also reviewed the references for pertinent articles.

### Article selection

We used Covidence software^39^ to manage the systematic review process. Two reviewers (L.U. and N.L.) performed the initial article screening by assessing titles and abstracts from the search. Article exclusion occurred if they lacked a GHE or virtual focus. After the initial exclusion process, L.U. and N.L. independently reviewed the full text of the remaining articles to determine whether articles met the predetermined eligibility criteria. Because the heterogeneity of articles included did not lend to formal quality evaluation, we jointly determined our parameters for making judgements and used three general ratings. “Good” and “fair” articles met inclusion criteria and included information on at least >75% or 50-75%, respectively, of planned data extraction points. “Poor” articles did not adequately meet inclusion criteria and/or did not contain sufficient information for data extraction. We included “good” articles, excluded “poor” articles, and further discussed “fair” articles to reach consensus. A third reviewer (S.K.L.) settled disagreements on inclusion or exclusion via collaborative consultation.

### Data extraction

Members of the study team independently extracted data from the articles in an Excel spreadsheet. Three reviewers (L.U., N.L. and N.E.H.) then cross-checked extracted data. Extracted data included: activity type, synopsis, ownership, length, frequency, content delivery, cost, evaluation, outcomes; targeted participant type, numbers, and location; drivers/enablers, barriers/challenges, and impact. We organized the VGHEAs into 8 activity types: synchronous activities (e.g., discussions, conferences, chats, skills sessions, simulations, or lectures); asynchronous activities (e.g., modules, videos, or pre-recorded lectures); group learning or projects; shared cloud resources; complete online GH courses; virtual mentorship; paired learning (“twinning”) experiences; and online discussion forums.

### Data synthesis and analysis

We performed a qualitative summary of the data given the nature of the systematic review and the preponderance of descriptive statistics in included papers. We summarized descriptive data, identified common collective themes, and noted gaps in available information.

## Results

**Figure 1:**
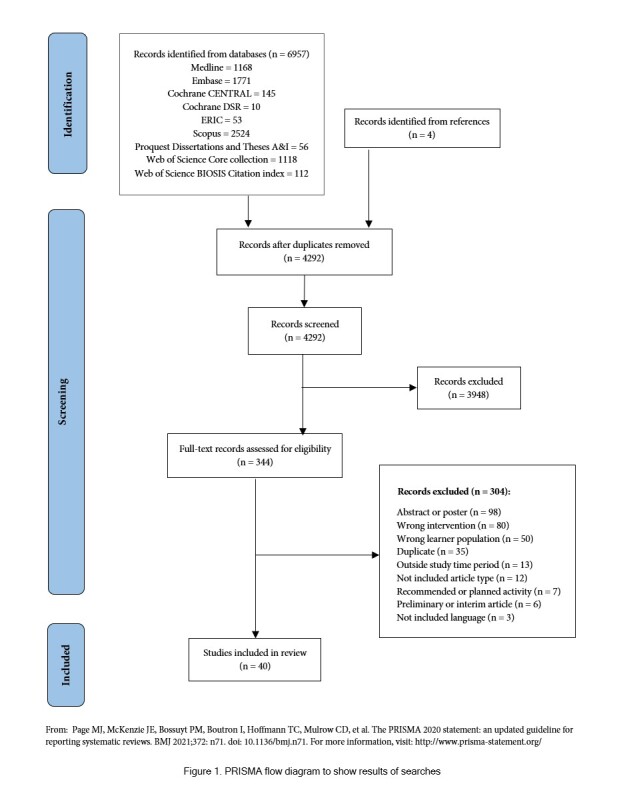
PRISMA flow diagram to show results of searches

Database searches identified a total of 6,957 references. Covidence removed 2,669 duplicates, leaving 4,288 citations for title and abstract screening. Forty articles were found to be of relevance to this review (Figure 1). 

### General descriptions of VGHEA articles

Table 1 provides general descriptions of the 40 included articles, including descriptions of 69 different VGHEAs. Many articles (48%, 19/40) described newly formed VGHEAs existing for < 1 year. The most common format of VGHEAs (25%, 10/40 of included papers) utilized regularly available online content or short courses in GH. Most articles (70%, 28/40) reported online-only activities, while 30% (12/40) reported hybrid or blended activities that included both online and in-person components. Most activities (48%, 19/40) were synchronous, 30% (12/40) were asynchronous, 17% (7/40) were both, and 5% (2/40) were downloadable materials only. Most activities (65%, 26/40) were available through a university, with smaller subsets being available through a GH partner (13%, 6/40) or via open access online (10%, 4/40). One article (3%, 1/40) reported requiring payment for the activity, and another (3%, 1/40) reported detailed activity cost information.

### Types of VGHEAs

Most included articles (57%, 23/40) described multiple VGHEAs. The VGHEA activity types are as follows: synchronous activities (93%, 47/40 of articles); asynchronous activities (35%, 14/40); group learning or projects (23%, 9/40 of articles); shared cloud resources (15%, 6/40 of articles); complete GH courses (15%, 6/40 of articles); virtual mentorship (10%, 4/40 of articles); twinning experiences (5%, 2/40 of articles); and online discussion forums (10%, 4/40 of articles).

### Topic/focus of VGHEA

The complete list of topics covered in the described VGHEAs are listed in Table 1. Most articles (68%, 27/40) focused on general GH topics (e.g., global health education, community health, or field experiences) while 32% (13/40) focused on GH topics linked to a medical specialty (e.g., anesthesia or surgical training in LMICs). While the vast majority (95%, 38/40) of articles focused on international GH, two articles (5%, 2/40) focused on local GH. One paper (3%, 1/40) had health equity and equitable partnerships as a key focus.

### Trainee audience

Approximately 8400 total trainees were described in the included articles; one study (3%, 1/40) included 6000 trainees, and the remaining papers reported 11-501 trainees (mean 84). Targeted trainees were graduate medical students (57%, 23/40 of articles) or mixed audiences of health professional trainees (students, residents, or fellows) (33%, 13/40 of articles). Most articles (53%, 21/40) targeted trainees in both LMIC and HIC, while remaining articles reported targeting LMIC (22%, 9/40) or HIC (25%, 10/40) trainees alone.

Overall, few articles (10%, 4/40) reported details about trainee characteristics and rates of activity completion. One article (3%, 1/40) documented dropout rate of trainees through duration of the program, another (3%, 1/40) reported a documented increased participation rate over a two-year period during the activity, and two papers (5%, 2/40) provided a comparison of participation rates between trainees from HIC versus LMIC.

### Evaluation and outcomes of VGHEAs

Most articles (90%, 36/40) discussed VGHEA evaluations. The most reported evaluation method was participant surveys (57%, 23/40 of articles). Different outcome measures discussed are available in Table 1, the most common being satisfaction with course, content, or teaching (60%, 24/40 of articles) and self-reported improvement in knowledge or skills (40%, 16/40 of articles). Detailed evaluation methods, however, were not a common feature of included articles.

### Ownership and hosting of VGHEAs

Articles described a total of 31 countries (45%, 14/31 LMIC and 55%, 17/31 HIC) as hosts of the VGHEA(s). Most articles (68%, 27/40) reported hosting of the VGHEA by an individual institution, most commonly one within a HIC (65%, 26/40). One paper (3%, 1/40) reported a LMIC (Mexico) as the sole host, and no articles reported shared hosting between LMIC/LMIC partners.

Regarding authorship of included papers, 55% (22/40) had only authors from HIC institutions while 45% (18/40) had authors from both HIC and LMIC institutions.

**Table 1:** Article characteristics and summary of virtual global health education activities

Article	Virtual Global Health Education Activity (VGHEA) Details		
Author, Year, Title	VGHEA Description	VGHEA Characteristics	VGHEA Geography
Addo-Atuah (2014)^66^: A Global Health Elective Course in a PharmD Curriculum	Activities: Online blackboard sessions; Virtual/online team projects Focus: International global health Topics: Global health, pharmacy, social determinants of health, grant writing Audience: Student Evaluation: Student survey	Length: 1-5 years Frequency: Weekly Content delivery: Blended/hybrid Online form: Asynchronous Cost: University sponsored Ownership: Single institution	Host: High-income country (HIC) (USA) Trainees: HIC (USA)
Ambrose (2017)^21^: Learning global health: a pilot study of an online collaborative intercultural peer group activity involving medical students in Australia and Indonesia	Activities: Small group e-learning; Online communication tools; Shared online documents Focus: International global health Topics: Infectious disease and trauma Audience: Student Evaluation: Student survey	Length: < 1 year Frequency: Short Course Content delivery: Online Online form: Mixed formats Cost: University sponsored Ownership: Unknown	Host: High-income country (HIC) and low-to middle income country (LMIC) (Australia and Indonesia) Trainees: HIC and LMIC (Australia and Indonesia)
Amerson (2019)^67^: Preparing Undergraduates for the Global Future of Health Care	Activities: Online materials; Virtual classroom Focus: International global health Topics: Social determinants of health, leadership in global health, field experiences Audience: Student Evaluation: Student survey	Length: 1-5 years Frequency: Biannually Content delivery: Blended/hybrid Online form: Mixed formats Cost: University sponsored Ownership: Single institution	Host: High-income country (HIC) (USA) Trainees: HIC (USA)
Atkins (2016)^56^: Student experiences of participating in five collaborative blended learning courses in Africa and Asia: a survey	Activities: Online lectures, readings, discussion forums Focus: International global health Topics: Pharmacology, health system evaluation, research Audience: Student Evaluation: Student and facilitator surveys	Length: 1-5 years Frequency: Unknown Content delivery: Blended/ hybrid Online form: Mixed formats Cost: University sponsored Ownership: Unknown	Host: High-income country (HIC) and low-to middle income country (LMIC) (India, China, Vietnam, Sweden, South Africa, Uganda, Malawi, China) Trainees: HIC and LMIC (India, China, Vietnam, Sweden, South Africa, Uganda, Malawi, China)
Bensman (2017)^57^: Creating Online Training for Procedures in Global Health with PEARLS (Procedural Education for Adaptation to Resource-Limited Settings)	Activities: Online videos and downloadable materials Focus: International global health Topics: Pediatric procedural skills Audience: Mixed audience Evaluation: Website data	Length: > 5 years Frequency: Available online content Content delivery: Blended/ hybrid Online form: Downloadable materials Cost: Open access Ownership: Multiple institution	Host: High-income country (HIC) (USA) Trainees: HIC and low-to middle income country (LMIC) (USA, various)
Bolon (2020)^46^: One Health education in Kakuma refugee camp (Kenya): From a MOOC to projects on real world challenges	Activities: Online peer-to-peer learning, lecturing, and mentoring; massive open online course (MOOC) Focus: International global health Topics: Public health problems Audience: Student Evaluation: Student and facilitator surveys and focus groups	Length: < 1 year Frequency: Short course Content delivery: Blended/hybrid Online form: Synchronous Cost: University sponsored Ownership: Multiple institution	Host: High-income country (HIC) (Switzerland) Trainees: Low-to middle income country (LMIC) (Kenya)
Bothara (2021)^47^: Global health classroom: mixed methods evaluation of an interinstitutional model for reciprocal global health learning among Samoan and New Zealand medical students	Activities: Videoconference classroom for case discussions Focus: International global health Topics: General public health Audience: Student Evaluation: Student survey	Length: 1-5 years Frequency: Weekly Content delivery: Blended/ hybrid Online form: Synchronous Cost: University sponsored Ownership: Multiple institution	Host: High-income country (HIC) (New Zealand) Trainees: HIC and low-to middle income country (LMIC) (New Zealand, Samoa)
Bowen (2021)^58^: Virtual Exchange in Global Health: an innovative educational approach to foster socially responsible overseas collaboration	Activities: Virtual student short-term experiences in global health Focus: International global health Topics: Refugee health, global health Audience: Student Evaluation: Student survey	Length: 1-5 years Frequency: Short course Content delivery: Online Online form: Synchronous Cost: University sponsored Ownership: Single institution	Host: High-income country (HIC) (USA) Trainees: HIC and low-to middle income country (LMIC) (USA, Lebanon)
Carrasco (2020)^64^: Evaluation of a multidisciplinary global health online course in Mexico	Activities: Online global health course (zoom sessions, team-based activities, reflective writing, and final project) Focus: Local global health Topics: Global health education and leadership Audience: Student Evaluation: Student survey	Length: 1-5 years Frequency: Biannually Content delivery: Online Online form: Synchronous Cost: University sponsored Ownership: Single institution	Host: Low- to middle-income country (LMIC) (Mexico) Trainees: Low-to middle income country (LMIC) (Mexico)
Chastonay (2015)^68^: A public health e-learning master's programme with a focus on health workforce development targeting francophone Africa: The University of Geneva experience	Activities: Online e-modules and distance learning via an electronic platform Focus: International global health Topics: Global health Audience: Mixed audience Evaluation: Student and facilitator surveys; course data, knowledge assessments; and community assessments	Length: 1-5 years Frequency: Short course Content delivery: Online Online form: Asynchronous Cost: University sponsored Ownership: Multiple institution	Host: High-income country (HIC) (Switzerland) Trainees: Low-to middle income country (LMIC) (Burkina Faso, Burundi, Cameroon, Chad, Central African Republic, Congo, DR Congo, Ivory Coast, Mali, Senegal)
DeCamp (2013)^25^: An ethics curriculum for short-term global health trainees	Activities: Online modules Focus: International global health Topics: Global health ethics Audience: Mixed audience Evaluation: Student survey and website data	Length: > 5 years Frequency: Available online content Content delivery: Online Online form: Downloadable materials Cost: Open access Ownership: Multiple institution	Host: High-income country (HIC) (USA) Trainees: HIC (USA)
Ezeonwu (2014)^63^: Using an academic-community partnership model and blended learning to advance community health nursing pedagogy	Activities: Online discussions Focus: Local global health Topics: Health screening and education Audience: Student Evaluation: Reflective writing	Length: 1-5 years Frequency: Weekly Content delivery: Blended/hybrid Online form: Asynchronous Cost: University sponsored Ownership: Single institution	Host: High-income country (HIC) (USA) Trainees: HIC (USA)
Falleiros de Mello (2018)^69^: An Innovative Exchange Model for Global and Community Health Nursing Education	Activities: Online presentations; twinning Focus: International global health Topics: Nursing interventions within public health system Audience: Student Evaluation: Student survey	Length: < 1 year Frequency: Short course Content delivery: Blended/hybrid Online form: Asynchronous Cost: University sponsored Ownership: Multiple institution	Host: High-income country (HIC) and low-to middle income country (LMIC) (USA, Brazil) Trainees: HIC and LMIC (USA, Brazil)
Gros (2021)^53^: Innovation in resident education -- Description of the Neurology International Residents Videoconference and Exchange (NIRVE) program	Activities: Online case presentations Focus: International global health Topics: Neurology images and case studies Audience: Resident Evaluation: None	Length: > 5 years Frequency: Monthly Content delivery: Online Online form: Synchronous Cost: Available through partners Ownership: Multiple institution	Host: High-income country (HIC) (Canada) Trainees: HIC and low-to middle income country (LMIC) (Various)
Gruner (2015)^70^: Introducing global health into the undergraduate medical school curriculum using an e-learning program: a mixed method pilot study	Activities: Online modules and case studies, videos, and resources Focus: International global health Topics: Refugee health and general global health Audience: Student Evaluation: Third-party evaluation	Length: < 1 year Frequency: Unknown Content delivery: Online Online form: Asynchronous Cost: University sponsored Ownership: Single institution	Host: High-income country (HIC) (Canada) Trainees: HIC (Canada)
Hannigan (2015)^71^: Sharing a Piece of the PIIE: Program of international interprofessional education/programa internacional interprofesional educativo	Activities: Online discussions Focus: International global health Topics: Primary care Audience: Student Evaluation: Student survey	Length: < 1 year Frequency: Unknown Content delivery: Online Online form: Synchronous Cost: Unknown Ownership: Multiple institution	Host: High-income country (HIC) and low-to middle income country (LMIC) (USA, Dominican Republic) Trainees: HIC and LMIC (USA, Dominican Republic)
Haynes (2021)^48^: Global Health Imperative to Prioritizing Cardiovascular Education	Activities: Online lectures, image sharing, discussions Focus: International global health Topics: Cardiology Audience: Mixed audience Evaluation: Student survey and qualitative analysis	Length: 1-5 years Frequency: Short course Content delivery: Online Online form: Mixed formats Cost: Unknown Ownership: Multiple institution	Host: High-income country (HIC) and low-to middle income country (LMIC) (USA, France, Haiti) Trainees: HIC and LMIC (USA, France, Haiti)
Hou (2020)^49^: Impact of the COVID-19 pandemic on global health research training and education	Activities: Online lectures, videos, role play and small group discussions; digital/virtual training sessions; virtual whiteboards Focus: International global health Topics: Cancer epidemiology and pathology and molecular lab medicine Audience: Mixed audience Evaluation: None	Length: < 1 year Frequency: Monthly Content delivery: Online Online form: Synchronous Cost: University sponsored Ownership: Multiple institution	Host: High-income country (HIC) (USA) Trainees: Low-to middle income country (LMIC) (Nigeria)
Jacquet (2018)^50^: The Practitioner's Guide to Global Health: an interactive, online, open-access curriculum preparing medical learners for global health experiences	Activities: Massive online open course (MOOC) Focus: International global health Topics: Preparation for short term experiences in global health Audience: Mixed audience Evaluation: Student survey and knowledge assessment	Length: 1-5 years Frequency: Unknown Content delivery: Online Online form: Asynchronous Cost: Open access Ownership: Multiple institution	Host: High-income country (HIC) and low-to middle income country (LMIC) (Canada, India, Kenya, Lebanon, Moldova, South Africa, United Kingdom, and USA) Trainees: HIC and LMIC (Various)
Jiang (2021)^40^: An International Virtual Classroom: The Emergency Department Experience at Weill Cornell Medicine and Weill Bugando Medical Center in Tanzania	Activities: Online lectures, mentoring, discussion forums and resource sharing; virtual collaboration on clinical protocol development Focus: International global health Topics: Emergency medicine Audience: Resident Evaluation: None	Length: < 1 year Frequency: Monthly Content delivery: Online Online form: Mixed formats Cost: University sponsored Ownership: Multiple institution	Host: High-income country (HIC) (USA) Trainees: Low-to middle income country (LMIC) (Tanzania)
Kiwanuka (2015)^59^: Synchronous distance anesthesia education by Internet videoconference between Uganda and the United States	Activities: Online lectures Focus: International global health Topics: Anesthesia Audience: Resident Evaluation: Knowledge assessment	Length: < 1 year Frequency: Weekly Content delivery: Online Online form: Synchronous Cost: University sponsored Ownership: Single institution	Host: High-income country (HIC) and low-to middle income country (LMIC) (USA, Uganda) Trainees: HIC and LMIC (USA, Uganda)
Krohn (2021)^54^: Global Health Education during the COVID-19 Pandemic: Challenges, Adaptations, and Lessons Learned	Activities: Online modules, group discussions; virtual lab sessions with digital microscopy Focus: International global health Topics: General global health Audience: Mixed audience Evaluation: Student survey	Length: < 1 year Frequency: Yearly Content delivery: Online Online form: Synchronous Cost: Paid Ownership: Single institution	Host: High-income country (HIC) (USA) Trainees: HIC and low-to middle income country (LMIC) (US, Panama, Thailand, Jordan, UK, Australia, New Zealand, Gabon, Kenya, Tunisia)
Kulier (2012)^72^: Effectiveness of a Clinically Integrated e-Learning Course in Evidence-Based Medicine for Reproductive Health Training	Activities: Online videos, modules, and self-guided sessions Focus: International global health Topics: Evidence-based medicine Audience: Resident Evaluation: Student survey and participant observation	Length: < 1 year Frequency: Unknown Content delivery: Blended/ hybrid Online form: Asynchronous Cost: Available through partners Ownership: Single institution	Host: High-income country (HIC) (UK) Trainees: Low-to middle income country (LMIC) (Argentina, Brazil, Democratic Republic of the Congo, India, Philippines, South Africa, Thailand)
Kuriyan (2014)^51^: Innovations in nutrition education and global health: the Bangalore Boston nutrition collaborative	Activities: Online supplemental learning to in-person learning; virtual mentorship Focus: International global health Topics: Nutrition Audience: Mixed Evaluation: Student and alumni surveys	Length: 1-5 years Frequency: Yearly Content delivery: Blended/ hybrid Online form: Asynchronous Cost: University sponsored Ownership: Multiple institution	Host: High-income country (HIC) and low-to middle income country (LMIC) (India, USA) Trainees: LMIC (India, Nepal, Pakistan, Bangladesh, Uganda)
Lee (2020)^73^: The feasibility and satisfaction of an online global health education course at a single medical school: a retrospective study	Activities: Online global health course Focus: International global health Topics: Introduction to global health Audience: Student Evaluation: Student survey, knowledge assessment, and course data	Length: < 1 year Frequency: Yearly Content delivery: Online Online form: Synchronous Cost: University sponsored Ownership: Single institution	Host: High-income country (HIC) (Korea) Trainees: HIC (Korea)
Martini (2012)^74^: Triune Case Study: An Exploration into Inter-Professional Education (IPE) in an Online Environment Supporting Global Health	Activities: Online lectures, chats, and discussions Focus: International global health Topics: Infectious diseases Audience: Student Evaluation: Student and facilitator surveys	Length: < 1 year Frequency: Unknown Content delivery: Online Online form: Mixed formats Cost: Available through partners Ownership: Multiple institution	Host: High-income country (HIC) and low-to middle income country (LMIC) (New Zealand, Australia, Uganda) Trainees: HIC and LMIC (Various)
Mirza (2021)^55^: Utilizing virtual exchange to sustain global health partnerships in medical education	Activities: Cloud-based case discussions and idea exchanges Focus: International global health Topics: Infectious diseases Audience: Mixed audience Evaluation: Student survey	Length: < 1 year Frequency: Monthly Content delivery: Online Online form: Synchronous Cost: Unknown Ownership: Unknown	Host: High-income country (HIC) (USA) Trainees: HIC and low-to middle income country (LMIC) (USA, China)
Poirier (2016)^75^: Interprofessional Online Global Health Course	Activities: Online small group assignments and discussions; asynchronous readings, quizzes, and self-study questions Focus: International global health Topics: Global health, interprofessional education Audience: Student Evaluation: Student survey, knowledge assessment, course data	Length: 1-5 years Frequency: Yearly Content delivery: Online Online form: Synchronous Cost: University sponsored Ownership: Single institution	Host: High-income country (HIC) (USA) Trainees: HIC (USA)
Prosser (2021)^60^: Reflective practice and transcultural psychiatry peer e-learning between Somaliland and the UK: a qualitative evaluation	Activities: Peer-to-peer e-learning Focus: International global health Topics: Mental health Audience: Student Evaluation: Reflective writing and focus groups	Length: 1-5 years Frequency: Weekly Content delivery: Online Online form: Synchronous Cost: University sponsored Ownership: Multiple institution	Host: High-income country (HIC) and low-to middle income country (LMIC) (UK, Somaliland) Trainees: HIC and LMIC (UK, Somaliland)
Ravi (2021)^76^: Fostering bidirectional trainee-led partnerships through a technology-assisted journal club - The GASOC experience	Activities: Online journal clubs Focus: International global health Topics: Global anesthesia, surgery obstetrics Audience: Mixed audience Evaluation: Student survey	Length: 1-5 years Frequency: Unknown Content delivery: Online Online form: Synchronous Cost: Open access Ownership: Multiple institution	Host: High-income country (HIC) and low-to middle income country (LMIC) (UK, South Africa, Ireland) Trainees: HIC and LMIC (Various)
Samuels (2020)^61^: Evaluation of the effectiveness of the Global Medical Student Partnership program in undergraduate medical education	Activities: Online case discussions Focus: International global health Topics: Naturopathic medicine; women’s health; medicine in conflict zones; palliative care; pandemics; mental health Audience: Student Evaluation: Student survey	Length: < 1 year Frequency: Monthly Content delivery: Online Online form: Mixed formats Cost: Unknown Ownership: Single institution	Host: High-income country (HIC) (Canada) Trainees: HIC and low-to middle income country (LMIC) (Canada, Ethiopia, Israel, Jamaica, Saudi Arabia)
Sarkar (2015)^77^: Community health nursing through a global lens C3 - Studies in Health Technology and Informatics	Activities: Virtual classrooms via videoconferencing Focus: International global health Topics: Social determinants of health Audience: Student Evaluation: Student and facilitator surveys	Length: > 5 years Frequency: Biannually Content delivery: Online Online form: Synchronous Cost: University sponsored Ownership: Single institution	Host: High-income country (HIC) (USA) Trainees: HIC and low-to middle income country (LMIC) (USA, Ecuador, India, Haiti)
Stallwood (2020)^65^: Applying equity-centered principles in an interprofessional global health course: a mixed methods study	Activities: Online lectures and group projects Focus: International global health Topics: Promotion of health equity equitable partnership development Audience: Mixed audience Evaluation: Student survey, interviews, and participant observation	Length: < 1 year Frequency: Weekly Content delivery: Blended/ hybrid Online form: Synchronous Cost: University sponsored Ownership: Unknown	Host: High-income country (HIC) (Canada) Trainees: HIC (Canada)
Sue (2018)^78^: The ReSurge Global Training Program: A Model for Surgical Training and Capacity Building in Global Reconstructive Surgery	Activities: Online modules Focus: International global health Topics: Reconstructive surgical techniques and education Audience: Mixed audience Evaluation: Facilitator survey	Length: 1-5 years Frequency: Monthly Content delivery: Blended/ hybrid Online form: Asynchronous Cost: Available through partners Ownership: Single institution	Host: High-income country (HIC) and low-to middle income country (LMIC) (Various) Trainees: LMIC (Vietnam, Ecuador, Nepal, Zimbabwe, Mozambique, Nicaragua, Bhutan, India, Bolivia, Cuba)
Taekman (2017)^62^: A Novel Multiplayer Screen-Based Simulation Experience for African Learners Improved Confidence in Management of Postpartum Hemorrhage	Activities: Virtual simulation Focus: International global health Topics: Post-partum hemorrhage management Audience: Mixed audience Evaluation: Student survey	Length: < 1 year Frequency: Short course Content delivery: Online Online form: Synchronous Cost: University sponsored Ownership: Single institution	Host: High-income country (HIC) (USA) Trainees: Low-to middle income country (LMIC) (Uganda)
Thorp (2021)^79^: WhatsApp Linking Lilongwe, Malawi to Los Angeles: Impacting Medical Education and Clinical Management	Activities: Consultations and case discussions via WhatsApp Focus: International global health Topics: Clinical case studies Audience: Mixed audience Evaluation: Student and facilitator surveys	Length: 1-5 years Frequency: As needed online interaction Content delivery: Online Online form: Synchronous Cost: University sponsored Ownership: Single institution	Host: High-income country (HIC) and low-to middle income country (LMIC) (USA, Malawi) Trainees: HIC and LMIC (USA, Malawi)
Ton (2015)^80^: The Development and Implementation of a Competency-Based Curriculum for Training in Global Health Research	Activities: Online modules, discussions lectures, mentorship; facilitated web conferencing; discussion boards Focus: International global health Topics: Global health research training Audience: Fellow Evaluation: Student survey and course data	Length: < 1 year Frequency: Yearly Content delivery: Online Online form: Asynchronous Cost: Available through partners Ownership: Multiple institution	Host: High-income country (HIC) (USA) Trainees: HIC and low-to middle income country (LMIC) (Various)
Utley-Smith (2017)^81^: An online education approach to population health in a global society	Activities: Online course with faculty discussions Focus: International global health Topics: Global health for graduate nurses Audience: Student Evaluation: Student survey	Length: < 1 year Frequency: Biannually Content delivery: Online Online form: Asynchronous Cost: University sponsored Ownership: Single institution	Host: High-income country (HIC) (USA) Trainees: HIC (USA)
Wu (2020)^82^: An International Partnership of 12 Anatomy Departments - Improving Global Health through Internationalization of Medical Education	Activities: Online projects and small group discussions Focus: International global health Topics: Anatomy, cross-cultural discussions Audience: Student Evaluation: Student survey	Length: > 5 years Frequency: Yearly Content delivery: Online Online form: Mixed formats Cost: University sponsored Ownership: Multiple institution	Host: High-income country (HIC) (Austria, Australia, Canada, Denmark, Finland, Germany, Japan, Taiwan, United Kingdom, United States) Trainees: HIC (Austria, Australia, Canada, Denmark, Finland, Germany, Japan, Taiwan, United Kingdom, United States)
Ziemba (2016)^52^: Using International Videoconferencing to Extend the Global Reach of Community Health Nursing Education	Activities: Videoconference discussions Focus: International global health Topics: Global health nursing Audience: Student Evaluation: Student survey	Length: < 1 year Frequency: Biannually Content delivery: Online Online form: Synchronous Cost: University sponsored Ownership: Single institution	Host: High-income country (HIC) (USA) Trainees: HIC and low-to middle income country (LMIC) (USA, Haiti)

HIC = high-income country; LMIC = low- to middle-income country

No papers had only authors from LMIC institutions. Among the 18 articles with a mixed author group, 78% (14/18) had more HIC than LMIC authors; 11% (2/18) had more LMIC than HIC authors; and 11% (2/18) had equal numbers of LMIC and HIC authors. 92% (37/40) of articles had a HIC first author, and 90% (36/40) had a HIC last author.

### Participation in VGHEAs

The 40 included articles described 66 countries (73%, 48/66 LMIC and 27%, 18/66 HIC) as having participated in the VGHEAs. A HIC (USA) was the most frequent consumer of VGHEAs, followed by India, the UK and Uganda.

### Drivers/enablers and barriers/challenges of VGHEAs

Most papers discussed drivers/enablers (93%, 37/40) and barriers/challenges (98%, 39/40) of VGHEAs (Figure 2, Panel A and B, respectively), which we grouped into 14 categories each. The most common drivers/enablers were strong course content and organization (40%, 16/40 of articles); peer interactions (38%, 15/40 of articles); and activity ease/flexibility (30%, 12/40 of articles). The most common barriers/challenges were challenges to online trainee engagement (unequal participation/engagement or lack of interest/motivation; 48%, 19/40 of articles); issues with virtual platforms/technology or internet connectivity problems (45%, 18/40 of articles); and challenges with time zones or course hours (33%, 13/40 of articles).

Unexpected impact of the course (positive or negative) and wider benefits noted:

Overall, 58% (23/40) of included articles cited a wider positive impact of the VGHEA beyond what was originally expected. Table 2 presents common themes, such as a wider reach than in-person activities, real world impact, improved existing GH partnerships and activities, and newly identified gaps in GH practices.

Notably, one article (3%, 1/40) cited unanticipated negative consequences of the VGHEA, specifically that uncertainties for ongoing funding and lack of foreign recognition of course credit were unexpected hardships for course participants.

## Discussion

To the best of our knowledge, this is the first systematic review to identify and synthesize the recent landscape of VGHEAs, including their enablers and barriers. The findings in this review identify gaps in the literature needing future study and illustrate important themes that GH educators should consider when planning and developing VGHEAs.

Most of the VGHEAs described no cost participation or content, but importantly, most articles implied that participation was linked to university tuition or membership or only available via a GH partnership. These findings highlight the difficulty in accessing VGHEAs should a learner not be affiliated with a university or formal GH program or partnership. Aside from one paper, 40 there was a paucity of information regarding specific costs of the activities, both in terms of host cost (e.g., technology infrastructure, platform subscriptions, salary support, etc.) and trainee costs (e.g., university fees, personal costs, cost of data plans or Wi-Fi to access, etc.). Because GH experiences are linked with increased awareness of health system costs and issues, and because decreased funding for GH activities could lead to negative consequences for education, partnerships, and collaboration (disproportionately affecting LMIC partners), 6, 40-43 more financial information about VGHEAs would be useful to inform the discussion on the costs and benefits of continuing in-person travel for GH activities versus shifting to virtual activities long-term.

In terms of ownership and hosting of VGHEAs, there was a notable lack of both shared hosting between LMIC partners and of LMIC institutions that had sole hosting/ownership of the activity. Regarding participation, the USA was overall the biggest consumer of activities reported, but it was unclear from papers discussing participation in VGHEAs by multiple countries what proportion of participants came from HIC versus LMIC settings. These findings raise multiple questions for future study regarding who is making decisions about content topics, target audiences, and goals of GH activities; whether virtual iterations of activities are appropriate for different audience types; and what barriers the HIC partner can alleviate for the LMIC partner.^40^ Regarding authorship, the vast majority of included papers reflected first and last authors from HIC institutions and an overall majority of HIC authors. Although this trend of unequal representation of LMIC authors in the GH literature is documented,44, 45 it is perhaps a call to colleagues involved in GH partnerships to ensure equal ownership and authorship of the VGHEA content and academic outputs.

**Figure 2, Panel A:**
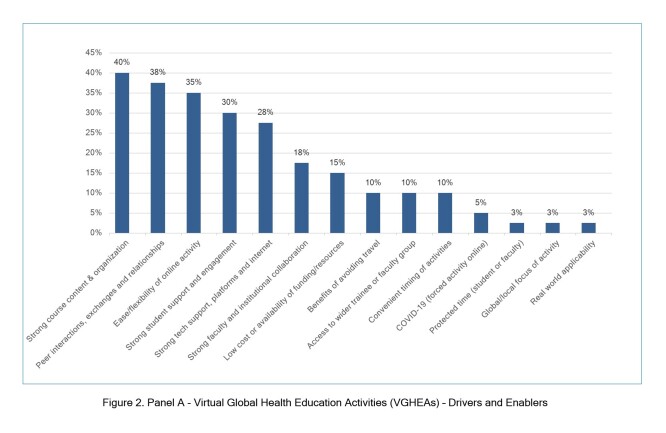
Virtual Global Health Education Activities (VGHEAs) – Drivers and Enablers

**Figure 2, Panel B:**
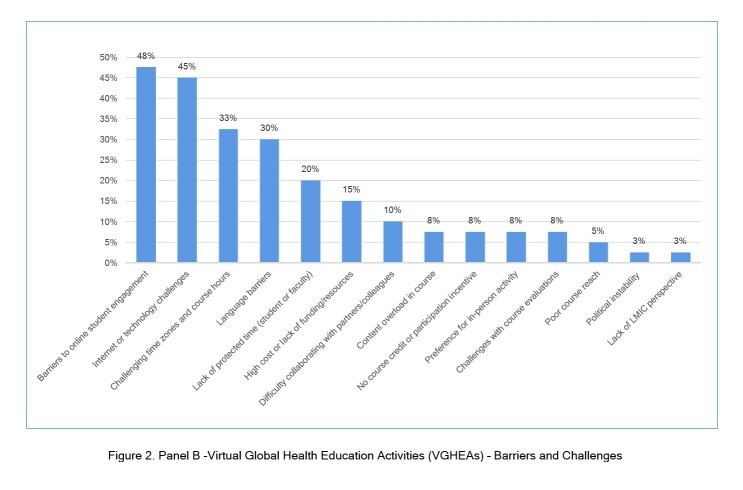
Virtual Global Health Education Activities (VGHEAs) – Barriers and Challenges

**Table 2:** Wider Positive Impacts of Virtual Global Health Education Activities

Positive Impact of Virtual Global Health Education Activities		Relevant Articles
Wider reach than in-person activities	Bridged geographic distance to bring education to those who may not otherwise have had access	Hou ^49^
	Facilitated a wider applicability of course content to other specialties or disciplines	Carrasco ^64^, Hou ^49^
	Improved understanding of importance and applicability of the virtual activity past the end of the COVID-19 pandemic	Bowen ^58^
	Facilitated a wider than anticipated reach of the VGHEA (such as with bigger trainee audiences, or better access to diverse faculty)	Hou ^49^
	Convenient, lower-cost, and more eco-friendly options for global health activities versus travel	Bowen ^58^, Gros ^53^, Samuels ^61^
Real World Impact	Increased clinical activities or capacities because of the activity	Sue ^78^
	Contributed to positive real-life impact on participants who took the course (such as improved skills or knowledge or concrete preparation for in-person activities)	Addo-Atuah ^66^, Carrasco ^64^, Chastonay ^68^
	Informed or directly contributed to expansion of VGHEA activity or content to other courses, global health activities, resources, or institutional departments	Addo-Atuah ^66^, Atkins ^56^, Bolon ^46^, Carrasco ^64^, Chastonay ^68^, Gros ^53^
	Led to career advancement or enrichment, both for faculty and trainees	Addo Atuah ^66^, Amerson ^67^, Bolon ^46^, Chastonay ^68^
Improved existing GH partnerships and activities	Improved relationships between participating faculty, institutions, or global health partners	Chastonay ^68^, Kiwanuka ^59^
	Led to spin-off projects, new partnership activities, or larger initiatives that had a positive impact on health	Bensmen ^57^, Chastonay ^68^, Ezeonwo ^63^, Kiwanuka ^59^, Martini ^74^
	Supplemented in-person activities positively and effectively	Ambrose ^21^, Bensman ^57^, Gruner ^70^, Samuels ^61^
Identified gaps in GH practices	Highlighted the need for regular or improved evaluation of an activity, particularly in terms of longer-term impacts	Atkins ^56^, Jacquet ^70^
	Highlighted the need to include an indigenous perspective in the activity	Stallwood ^70^

VGHEA = virtual global health education activity

Regarding targeted audiences, our team found surprisingly minimal information about the trainees in the included papers. Further elucidation of learner types and geographic distribution would be key in future studies to better understand activity uptake and appropriateness, particularly for unique LMIC learners, such as in refugee settings. 46 We also found that HIC audiences made up a larger proportion of targeted trainees. This merits further discussion in terms of how much content should be directed toward HIC consumers (specifically when the education is preparing for HIC trainees for experiences in LMIC settings) versus content focusing on building support for LMIC partners and addressing health disparities.

Included articles discussed VGHEA evaluations and measured various outcomes, but details about the evaluation methods were not always well described, nor were outcomes standard even among similar activities. Key gaps in our included literature sample appear to be standard evaluation tools, how to best document VGHEA effectiveness, and critically, how the VGHEA affects relevant communities after trainees completed the activity. Documenting and exploring these topics could have large implications for GH educators seeking concrete guidance on best practices for VGHEA evaluation and quality improvement.

The several enablers and barriers of VGHEAs and key themes identified provide important considerations for GH educators. Certain elements were both enablers and barriers, specifically funding, the need for protected and convenient course timing, and technological support needed for VGHEA implementation. The double mention of these factors highlights their critical importance to the success of VGHEAs; indeed, those articles that mentioned funding,^47^^-^^52^ timing,^40^^, ^^53^^-^^55^ and strong technology^40^^, ^^49^^, ^^53^^, ^^54^^, ^^56^^-^^62^ as facilitators of VGHEAs offer key insights into how to overcome barriers that may prevent successful VGHEA implementation. More research in this area will be important to guide the planning and development of VGHEAs, particularly between HIC/LMIC partners who will have different needs and capacities.

Our group noted several gaps in the available literature that could benefit from future study to better guide GH educators in their virtual program planning. In terms of the VGHEAs described in the 40 unique articles, we found that most papers provided basic, descriptive information only. While this information is useful to document the current landscape of VGHEAs, there was less information regarding best practice recommendations for described activities, specifically in terms of frequency, evaluation, duration, organization, and content. Additionally, included articles addressed a wide range of VGHEAs covering multiple topics. Further discussion is warranted on what types of activities work best in certain contexts and for which type of trainees. Virtual domestic or global-local activities, an important subject mentioned in only two articles,^63^^,^^64^ likewise merits future discussion. Last, there was a dearth of information on sustained virtual engagements to benefit ongoing GH partnerships, particularly for partners in LMICs. Only one paper 65 mentioned health equity and equitable partnerships as a topic area, specifically suggesting the need to have an indigenous perspective included in the course presentation. In the future, it will be important to discuss who decides on the topics included in each activity, particularly for those in LMIC consuming material made by HIC educators. Over the coming years, these considerations may influence virtual GHE planning and implementation at graduate medical institutions worldwide.

Our review had several limitations. First, authors attempted to identify all relevant VGHEA articles, but many initiatives prompted by the pandemic were most likely underway but not yet published. Second, we only included VGHEAs focusing on health professional trainees; future investigation into how community health workers or health professions engage with VGHEAs could be of benefit. Third, although the grey literature search found no additional articles to be screened after cross-referencing article databases and online repositories, we found but excluded an abundance of GH activities (typically on websites, in conference proceedings and abstracts, and on online discussion forums) without a link to primary literature; a future mapping of these resources would be useful. Lastly, the broad nature of GHE introduces the possibility of bias in how we defined an activity and decided on inclusion. Addressing these limitations in future reviews would further contribute to guidelines for graduate GH educators.

## Conclusions

Our systematic review is the first review to identify and synthesize recent VGHEAs and report on the drivers and barriers that exist in the current literature. The field of VGHEA remains heterogenous and few studies aimed to examine best practices in the development of VGHEA. With medical trainees from HIC being the primary consumer of VGHEA, further consideration on how to be meet the needs of LMIC trainees is needed. These insights may provide guidance to GH educators in their planning and implementation of VGHEAs moving forward. Further work is needed on activity preferences, considerations for LMIC learners, best practice recommendations, and how activities could be created, shared, and consumed more equitably by partners from both HIC and LMIC settings. This review contributes meaningful foundational data to guide discussions among GH educators to address these knowledge gaps.

### Acknowledgements

The authors wish to thank Geoffrey Winstanley for his assistance in the creation of included figures.

### Conflict of Interest

The authors declare that they have no conflict of interest.

## Supplementary material

Supplementary file 1 Appendix 1 Virtual global health education activity review inclusion and exclusion criteria

Supplementary file 2 Appendix 2 Complete search strategies
