# Supplementary material for: Virtual global health in graduate medical education: a systematic review
Source: Int J Med Educ. 2022 Aug 31;13:230–48. doi: 10.5116/ijme.62eb.94fa (PMC9911141; doi:10.5116/ijme.62eb.94fa)
Supplement: Supplementary file 1 — Appendix 1 Virtual global health education activity review inclusion and exclusion criteria [file ijme-13-230-S1.pdf]

Appendix 1

Virtual global health education activity review inclusion and exclusion criteria

Inclusion

- Articles in English, Spanish, or French
- Content of *curriculum/program/activity/content* must have a global health focus, meaning must include recognition of disparity
- in resource level
- The topic of global health *curriculum/program/activity/content* must be focused on research, clinical, or public health work
- A primary focus of the article is on the domestic or international administration of global health *curricula/programs/activities/content*
- *Curriculum/program/activity/content* must be primarily administered virtually (synchronously or asynchronously online, by phone, or by web-based application), not primarily as an in-person activity with a supplementary and secondary virtual component
- Target users of *curriculum/program/activity/content* are any level trainee in any pre-professional or postgraduate medical specialty
- *Curriculum/program/activity/content* must involve regular, longitudinal, and/or ongoing global health activities, not isolated one-time events

Exclusion

- Articles prior to 2012 (included 10-year window from 2012-2021)
- Planned or recommended virtual global health education activities
- Posters from conferences
- Abstracts for which the associated PDF full text article is unavailable
- Theses, dissertations or book chapters
- Websites with global health education content not otherwise described in the primary literature
- Commentaries on or letters to the editor about virtual global health activities
- Duplicate articles (either exact duplicates or similar duplicates in different journals)
- Interim articles with a later more complete articles; will only include reports on studies with the most patient included and/or the longest follow-up times
- Isolated global health education activities not a part of a larger program or experience (i.e., a single journal club, mentorship on 1 medical student during one away rotation, etc.)
- *Curriculum/program/activity/content* focusing on:
    - Community health workers
    - The ECHO program or on ECHO program audiences
    - Rural healthcare providers linked to a larger health system without a focus on resource disparities between the rural and referral sites.
    - General medical education without a global health focus
    - Open access online content without a stated objective to reach trainees in under-resourced or LMIC settings
    - Telemedicine or tele-consult services without a stated objective to provide education or mentorship to trainees in under-resourced or LMIC settings
    - Non-human global health topics (i.e., veterinary care)
    - Trainees outside of pre-professional or postgraduate medical specialties (including biomedical or engineering trainees)
    - Continuing medical education focus directed at professionals who already completed previous training
