# Supplementary material for: Virtual global health in graduate medical education: a systematic review
Source: Int J Med Educ. 2022 Aug 31;13:230–48. doi: 10.5116/ijme.62eb.94fa (PMC9911141; doi:10.5116/ijme.62eb.94fa)
Supplement: Supplementary file 2 — Appendix 2 Complete search strategies [file ijme-13-230-S2.pdf]

Appendix 2

Complete search strategies

All searches originally run January 15, 2021 and updated on November 4, 2021. A combination of indexing terms and keywords was used to capture the concepts of either global health or tropical medicine with health professional education and modes of virtual learning. Explosion of indexing terms was utilized when appropriate. Along with truncation, the Boolean operator "OR" was used to capture alternate expressions of similar concepts to increase retrieval of potentially relevant citations.

Ovid MEDLINE® ALL <1946 to January 14, 2021>

updated: Ovid MEDLINE® ALL 1946 to November 03, 2021
1 Global Health/ or Tropical Medicine/ or (global health or world health or worldwide health or tropical medicine or international health or community health or bidirectional).tw.
2 exp education, dental/ or exp education, medical/ or exp education, nursing/ or exp education, pharmacy/ or exp education, public health professional/ or exp Health Education/ or exp Curriculum/ or International Educational Ex-change/
3 (dental education or medical education or nursing education or pharmacy education or pharmaceutical education or health professional education or health education).tw.
4 2 or 3
5 (1 and 4) or (Global Health/ed or Tropical Medicine/ed)
6 ((global health or world health or worldwide health or tropical medicine or international health or community health or bidirectional) adj3 (curricul* or education or training or communication or exchange* or partnership* or program* or collaboration)).tw.
7 5 or 6
8 (Internet-Based or Web-based or Online or Internet or virtual or telemedicine or e-learn* or eLearn* or electronic learning or Zoom or Skype or Facetime or digital platform or massive open online course* or MOOC* or distance education or videoconferenc* or web link* or web search*).tw.
9 Internet-Based Intervention/ or exp Telemedicine/ or Education, Distance/ or exp Videoconferencing/ or exp Online Systems/
10 8 or 9
11 7 and 10

Embase <1974 to 2021 January 14>

updated: Embase 1974 to 2021 November 03
1 global health/ or tropical medicine/ or (global health or world health or worldwide health or tropical medicine or international health or community health or bidirectional).tw.
2 exp dental education/ or exp medical education/ or exp nursing education/ or exp health education/ or exp curricu-lum/ or International Educational Exchange/
3 (dental education or medical education or nursing education or pharmacy education or pharmaceutical education or health professional education or health education).tw.
4 2 or 3
5 1 and 4
6 ((global health or world health or worldwide health or tropical medicine or international health or community health or bidirectional) adj3 (curricul* or education or training or communication or exchange* or partnership* or program* or collaboration)).tw.
7 5 or 6
8 (Internet-Based or Web-based or Online or Internet or virtual or telemedicine or e-learn* or eLearn* or electronic learning or Zoom or Skype or Facetime or digital platform or massive open online course* or MOOC* or distance education or videoconferenc* or web link* or web search*).tw.
9 web-based intervention/ or exp telemedicine/ or exp videoconferencing/ or exp online system/
10 8 or 9
11 7 and 10

ERIC - Education Resources Information Center (EBSCO interface)

S1: TI "global health" OR AB "global health" OR TI "world health" OR AB "world health" OR TI "worldwide health" OR AB "worldwide health" OR TI "tropical medicine" OR AB "tropical medicine" OR TI "international health" OR AB "interna-tional health" OR TI "community health" OR AB "community health" OR TI bidirectional OR AB bidirectional
S2: DE "Medical Education" OR DE "Graduate Medical Education" OR DE "Nursing Education" OR DE "Pharmaceutical Education" OR DE "Veterinary Medical Education" OR DE "Health Education" OR DE "Comprehensive School Health Education" OR DE "Curriculum" OR DE "Area Studies" OR DE "College Curriculum" OR DE "Continuous Progress Plan" OR DE "Core Curriculum" OR DE "Courses" OR DE "Elementary School Curriculum" OR DE "English Curriculum" OR DE "Ethnic Studies" OR DE "Experimental Curriculum" OR DE "Fused Curriculum" OR DE "Home Economics" OR DE "Honors Curriculum" OR DE "Integrated Curriculum" OR DE "Mathematics Curriculum" OR DE "Military Science" OR DE "Mod-ern Language Curriculum" OR DE "National Curriculum" OR DE "Preschool Curriculum" OR DE "Religion Studies" OR DE "Science Curriculum" OR DE "Secondary School Curriculum" OR DE "Shop Curriculum" OR DE "Social Studies" OR

DE "Speech Curriculum" OR DE "Spiral Curriculum" OR DE "Student Centered Curriculum" OR DE "Television Curricu-lum" OR DE "Unified Studies Curriculum" OR DE "Urban Studies" OR DE "Womens Studies" OR DE "International Educational Exchange"
S3: TI "dental education" OR AB "dental education" OR TI "medical education" OR AB "medical education" OR TI "nursing education" OR AB "nursing education" OR TI "pharmacy education" OR AB "pharmacy education" OR TI "Pharmaceutical Education" OR AB "Pharmaceutical Education" OR TI "health professional education" OR AB "health professional education" OR TI "health education" OR AB "health education"
S4: S2 OR S3
S5: S1 AND S4
S6: TX ("global health" OR "world health" OR "worldwide health" OR "tropical medicine" OR "international health" OR "community health" OR bidirectional) N3 (curricul* OR education OR training OR communication OR exchange* OR partnership* OR program* OR collaboration)
S7: S5 OR S6
S8: DE "Web Based Instruction" OR DE "Videoconferencing" OR DE "Distance Education" OR DE "Online Systems" OR DE "Interactive Video" OR DE "Online Catalogs" OR DE "Virtual Classrooms" OR DE "Online Courses" OR DE "Electron-ic Learning"
S9: TI (internet-based OR web-based OR online OR internet OR telemedicine or e-learn* or eLearn* OR "electronic learning" OR Zoom OR Skype OR Facetime OR "digital platform" OR "massive open online course*" OR MOOC* OR "distance education" OR videoconferenc* OR "web link*" OR "web search*") OR AB (internet-based OR web-based OR online OR internet OR telemedicine or e-learn* or eLearn* OR "electronic learning" OR Zoom OR Skype OR Facetime OR "digital platform" OR "massive open online course*" OR MOOC* OR "distance education" OR videoconferenc* OR "web link*" OR "web search*")
S10: S8 OR S9
S11: S7 AND S10

Web of Science Core Collection - all platforms/indexes:

-Science Citation Index Expanded (SCI-EXPANDED) --1965-present
-Social Sciences Citation Index (SSCI) --1965-present
-Arts & Humanities Citation Index (A&HCI) --1975-present
-Conference Proceedings Citation Index- Science (CPCI-S) --1990-present
-Conference Proceedings Citation Index- Social Science & Humanities (CPCI-SSH) --1990-present
-Emerging Sources Citation Index (ESCI) --2015-present
and
-BIOSIS Citation Index (BCI) --1969-2021
#1 - TOPIC=("global health" OR "world health" OR "worldwide health" OR "tropical medicine" OR "international health" OR "community health" OR bidirectional)
#2 - TOPIC: ("dental education" OR "medical education" OR "nursing education" OR "pharmacy education" OR "pharma-ceutical education" OR "Health professional education" OR "health education")
#3 - #1 AND #2
#4 - TOPIC=(("global health" OR "world health" OR "worldwide health" OR "tropical medicine" OR "international health" OR "community health" OR bidirectional) NEAR/3 (curricul* OR education OR training OR communication OR ex-change* OR partnership* OR program* OR collaboration))
#5 - #3 OR #4
#6 - TOPIC: (internet-based OR web-based OR online OR internet OR virtual OR telemedicine OR e-learn* OR eLearn* OR "electronic learning" OR Zoom OR Skype OR Facetime OR "digital platform" OR "massive open online course*" OR MOOC* OR "distance education" OR videoconferenc* OR "web link*" OR "web search*")
#7 - #5 AND #6

Scopus

1 - TITLE-ABS-KEY ("global health" OR "world health" OR "worldwide health" OR "tropical medicine" OR "interna-tional health" OR "community health" OR bidirectional)
2 - TITLE-ABS-KEY ("dental education" OR "medical education" OR "nursing education" OR "pharmacy education" OR "pharmaceutical education" OR "Health professional education" OR "health education")
3 - 1 AND 2
4 - TITLE-ABS-KEY (("global health" OR "world health" OR "worldwide health" OR "tropical medicine" OR "interna-tional health" OR "community health" OR bidirectional) W/3 (curricul* OR education OR training OR communi-cation OR exchange* OR partnership* OR program* OR collaboration))
5 - 3 OR 4
6 - (TITLE-ABS-KEY (internet-based OR web-based OR online OR internet OR virtual OR telemedicine OR e-learn* OR elearn* OR "electronic learning" OR zoom OR skype OR facetime OR "digital platform" OR "massive open online course*" OR mooc* OR "distance education") OR TITLE-ABS-KEY (videoconferenc* OR "web link*" OR "web search*"))
7 - 5 AND 6

Cochrane

Issue 1 of 12, January 2021
Cochrane Database of Systematic Reviews = 9
(1 editorial was yielded but there was no export option in Cochrane: https://doi-org.ccmain.ohionet.org/10.1002/14651858.ED000070)
Cochrane Central Register of Controlled Trials = 116

updated: Cochrane Central Register of Controlled Trials =141
Issue 10 of 12, October 2021
Cochrane Database of Systematic Reviews = 10
#1 MeSH descriptor: [Global Health] this term only
#2 MeSH descriptor: [Tropical Medicine] this term only
#3 ("global health" or "world health" or "worldwide health" or "tropical medicine" or "international health" or "community health" or bidirectional):ti,ab,kw
#4 #1 OR #2 OR #3
#5 MeSH descriptor: [Education, Dental] explode all trees
#6 MeSH descriptor: [Education, Medical] explode all trees
#7 MeSH descriptor: [Education, Nursing] explode all trees
#8 MeSH descriptor: [Education, Pharmacy] explode all trees
#9 MeSH descriptor: [Education, Public Health Professional] explode all trees
#10 MeSH descriptor: [Health Education] explode all trees
#11 MeSH descriptor: [Curriculum] explode all trees
#12 MeSH descriptor: [International Educational Exchange] this term only
#13 ("dental education" or "medical education" or "nursing education" or "pharmacy education" or "pharmaceutical education" or "health professional education" or "health education"):ti,ab,kw
#14 28-#13
#15 MeSH descriptor: [Global Health] explode all trees and with qualifier(s): [education - ED]
#16 MeSH descriptor: [Tropical Medicine] explode all trees and with qualifier(s): [education - ED]
#17 (("global health" or "world health" or "worldwide health" or "tropical health" or "international health" or "community health" or bidirectional) NEAR/3 (curricul* or education or training or communication or exchange* or partnership* or program* or collaboration)):ti,ab,kw
#18 (#4 AND #14) OR #15 OR #16 OR #17
#19 (Internet-Based or Web-based or Online or Internet or virtual or telemedicine or e-learn* or eLearn* or "elec-tronic learning" or Zoom or Skype or Facetime or "digital platform" or "massive open online course*" or MOOC* or "distance education" or videoconferenc* or "web link*" or "web search*"):ti,ab,kw
#20 MeSH descriptor: [Internet-Based Intervention] this term only
#21 MeSH descriptor: [Telemedicine] explode all trees
#22 MeSH descriptor: [Education, Distance] this term only
#23 MeSH descriptor: [Videoconferencing] explode all trees
#24 MeSH descriptor: [Online Systems] explode all trees
#25 28-#24
#26 #18 AND #25

ProQuest Dissertations & Theses A&I

S1 ti("global health" OR "world health" OR "worldwide health" OR "tropical medicine" OR "international health" OR "community health" OR bidirectional) OR ab("global health" OR "world health" OR "worldwide health" OR "tropical medicine" OR "international health" OR "community health" OR bidirectional)
S2 ti("dental education" OR "medical education" OR "nursing education" OR "pharmacy education" OR "pharmaceutical education" OR "health professional education" OR "health education") OR ab("dental education" OR "medical education" OR "nursing education" OR "pharmacy education" OR "pharmaceutical education" OR "health professional education" OR "health education")
S3 S1 AND S2
S4 ti(("global health" OR "world health" OR "worldwide health" OR "tropical medicine" OR "international health" OR "community health" or bidirectional) NEAR/3 (curricul* or education or training or communication or exchange* or partnership* or program* or collaboration)) OR ab(("global health" OR "world health" OR "worldwide health" OR "tropical medicine" OR "international health" OR "community health" or bidirectional) NEAR/3 (curricul* or education or training or communication or exchange* or partnership* or program* or collaboration))
S5 S3 OR S4
S6 ti(internet-based OR web-based OR online OR internet OR telemedicine OR e-learn* OR eLearn* OR "electronic learning" OR Zoom OR Skype OR Facetime OR "digital platform" OR "massive open online course*" OR MOOC* OR "distance education" OR videoconferenc* OR "web link*" OR "web search*") OR ab(internet-based OR web-based OR online OR internet OR telemedicine OR e-learn* OR eLearn* OR "electronic learning" OR Zoom OR Skype OR Facetime OR "digital platform" OR "massive open online course*" OR MOOC* OR "distance education" OR videoconferenc* OR "web link*" OR "web search*")
S7 S5 AND S6

Google grey-literature search strategy

Grey literature searched included Google search, CORE, OpenGrey, GreyNet International, Science.gov, WHO Interna-tional Global Health Observatory, WorldWideScience, Web of Conferences, the New York Academy of Medicine Grey Literature Report, and Duke University Grey Literature guide.

The following search terms were used to consider activities available through a university or school that was yet to be published.

- "site:.edu" google search and results
- "site:.edu global health education"
- "site:.edu global health education virtual"
- "site:.edu virtual global health"
